# Comatose With Basilar Artery Occlusion: Still Odds of Favorable Outcome With Recanalization Therapy

**DOI:** 10.3389/fneur.2021.665317

**Published:** 2021-05-04

**Authors:** Juhani Ritvonen, Tiina Sairanen, Heli Silvennoinen, Pekka Virtanen, Oili Salonen, Perttu J. Lindsberg, Daniel Strbian

**Affiliations:** ^1^Clinical Neurosciences, University of Helsinki, Helsinki, Finland; ^2^Neurological Research Unit, Department of Neurology, Neurocenter, Helsinki University Hospital, Helsinki, Finland; ^3^Helsinki Medical Imaging Center, Helsinki University Hospital, University of Helsinki, Helsinki, Finland

**Keywords:** basilar artery, outcome, coma, stroke, recanalization therapy

## Abstract

**Background:** Around 30–60% of patients with basilar artery occlusion (BAO) present with coma, which is often considered as a hallmark of poor prognosis.

**Aim:** To examine factors that will help predict outcomes in patients with BAO comatose on admission.

**Methods:** A total of 312 patients with angiography-proven BAO were analyzed. Comas were assessed as Glasgow Coma Scale (GCS) of ≤8 or impaired level of consciousness ascertained in the medical records. Outcomes were evaluated with the modified Rankin Scale (mRS) over a phone call at 3 months. In our study, 53 patients were excluded due to inadequate data on the level of consciousness.

**Results:** In total, 103/259 (39.8%) of BAO patients were comatose on admission. Factors associated with acute coma were higher age, coronary artery disease, convulsions, extent of early ischemia by posterior circulation Acute Stroke Prognosis Early CT Score (pc-ASPECTS) < 8, absence of patent posterior collateral vasculature, and occlusion over multiple segments of BA. A total of 21/103 (20.4%) of comatose patients had a favorable outcome (mRS 0–3), and 12/103 (11.7%) had a good outcome (mRS 0–2). Factors associated with a favorable outcome in comatose BAO patients were younger age (*p* = 0.010), less extensive baseline ischemia (*p* = 0.027), recanalization (*p* = 0.013), and avoiding symptomatic intracranial hemorrhage (sICH) (*p* = 0.038). Factors associated with the poorest outcome or death (mRS 5–6) were older age (*p* = 0.001), diabetes (*p* = 0.022), atrial fibrillation (*p* = 0.016), lower median GCS [4 (IQR 3.6) vs. 6 (5–8); *p* = 0.006], pc-ASPECTS < 8 (*p* = 0.003), unsuccessful recanalization (*p* = 0.006), and sICH (*p* = 0.010). Futile recanalization (mRS 4–6) was significantly more common in comatose patients (49.4 vs. 18.5%, *p* < 0.001).

**Conclusions:** One in five BAO patients with acute coma had a favorable outcome. Older patients with cardiovascular comorbidities and already existing ischemic lesions before reperfusion therapies tended to have a poor prognosis, especially if no recanalization is achieved and sICH occurred.

## Introduction

Basilar artery occlusion (BAO) accounts for 1% of all ischemic strokes but is still often considered the most appalling form of stroke (1, 2). The clinical picture of BAO varies greatly, and roughly 30–60% of patients present with the most shocking state, being comatose on admission (3–10). This initial coma or imminent locked-in state results typically from dense pontine ischemia, which anatomically encompasses essential areas forming the reticular activating system responsible for sustaining consciousness and motor pyramidal tracts (1, 11).

Indeed, in the face of the poor outcome reported to be associated with progressing pontine ischemia and depressed consciousness, acute coma in BAO, a discrete clinical entity, is one of the most challenging emergencies to be coped with by the emergency department (ED) attending neurologists and other acutologists (6, 12). However, it has also been reported that, with successful recanalization therapy, up to 15–26% of these patients presenting comatose on admission will eventually have a favorable outcome [modified Rankin Scale (mRS) 0–3] (6, 13, 14).

Which clinical signs, imaging characteristics and presumptive biomarkers could be used to assist in early risk stratification supporting initial management decisions, i.e., aggressive or de-escalated supportive therapy, and guide pressing interactions with the close ones? Mortality among intubated and mechanically ventilated stroke patients is high, yet the treatment decisions are not simple. It is one of the greatest challenges in the acute setting to estimate the prognostic potential to life “worth living” and the extent of treatments worth giving (15). Coma on presentation has been one negative prognostic marker to influence such decisions, especially regarding invasive therapies (4). Neurologists managing these patients often lack urgent directions in making these fundamental care decisions, for which this analysis was performed.

BAO carries high mortality of up to 95% if recanalization does not occur (1, 2, 7). Pallesen et al. (6) reported brainstem ischemia to be an important factor in predicting mortality in comatose BAO patients, which underlines the need for the early reversal of BAO with intravenous thrombolysis (IVT) or endovascular treatment (EVT). However, according to our knowledge, the baseline characteristics potentially predicting the outcome after BAO patients presenting with coma have never been systemically examined. This led us to carry out the present investigation in our sizable consecutive cohort of BAO patients treated with IVT and/or EVT.

## Aim of the Study

We set out to examine factors that would help in predicting the outcome in BAO patients presenting with acute coma on admission.

## Methods

### Patients

A total of 312 consecutive patients with angiography-proven BAO, treated between June 1995 and September 2019 in the Department of Neurology, Helsinki University Hospital, were analyzed. Coma was assessed as Glasgow Coma Scale (GCS) of ≤8 either in emergency medical service (EMS) records or at ED (whichever is lowest) before initiation of recanalization therapy. In subjects where GCS was not routinely reported, the level of consciousness was ascertained from the medical records describing the detailed responsiveness of the patient. In essence, the patient was defined as “comatose” if the level of consciousness was documented to be clearly impaired or if there was a need for intubation due to a low level of consciousness. We do performed endovascular intervention in the majority of the cases under conscious sedation, and if a patient was intubated only to perform neurointerventional therapy or neuroimaging, and not because of the low level of consciousness, the patient was not defined as “comatose.” As the level of consciousness on presentation could not be ascertained reliably due to insufficient EMS records and/or patient medical records, 53 patients were excluded.

Patient data, including also the 3-month follow-up assessment, were collected as a part of routine hospital care, and approval from an ethics committee or informed consent were thus not required to collect and reconcile retrospective clinical data in our institution. National Institutes of Health Stroke Scale (NIHSS) score is obtained for all acute recanalization patients on admission but since its determination may be interfered with by a low level of consciousness and possible anesthetic sedation, we excluded it from the analyses. The BAO “phenotype” was defined as a sudden or progressive onset of symptoms, as previously described (1).

### Imaging

**Figure 1:**
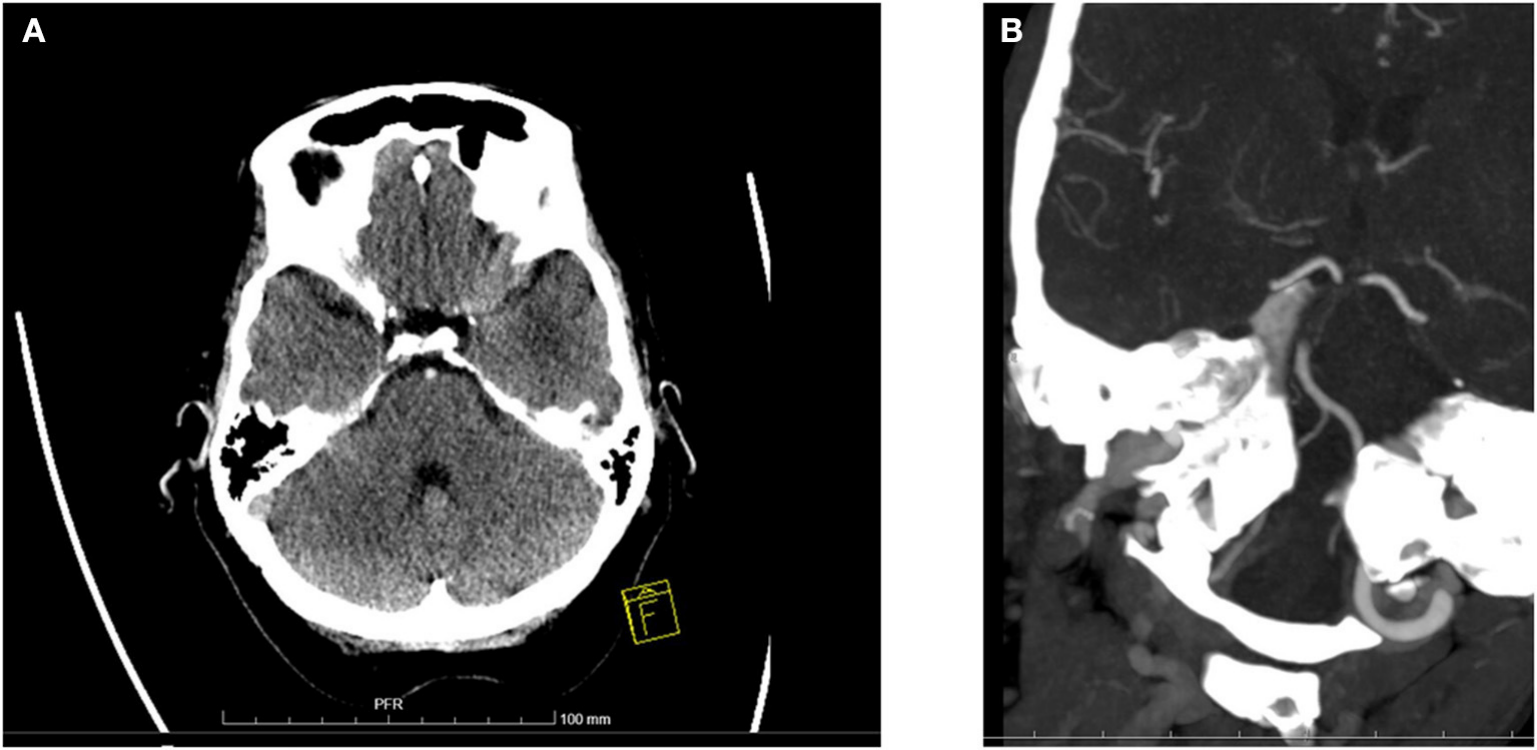
Baseline ischemic changes and basilar artery occlusion in computer tomography. **(A)** A 69-year-old woman was comatose on admission (GCS 4) and diagnosed with basilar artery occlusion. This baseline CT scan shows extensive ischemic changes in both cerebellar hemispheres and bilaterally in pons. Overall pc-ASPECTS was 5. A dense basilar artery is also seen. Patient received IVT but died on day 3 of hospitalization. **(B)** A 65-year-old woman was comatose on admission (GCS 6). Baseline CTA shows long BAO over mid- and caudal BA. IVT was administered and subsequent mechanical thrombectomy was performed with successful recanalization. Patient recovered with a 3-month mRS of 1.

Imaging was performed on admission for all patients with either computed tomography (CT) or magnetic resonance imaging (MRI) accompanied by CT angiography (CTA), MR angiography (MRA), or digital subtraction angiography (DSA). Control imaging was obtained ~24 h after treatment and whenever clinical deterioration occurred. The extent of baseline ischemia was evaluated with the posterior circulation Acute Stroke Prognosis Early CT Score (pc-ASPECTS) (16) (Figure 1).

Occlusion length was dichotomized to either “long,” consisting of two or more basilar artery segments (proximal, middle, and distal), or “short,” consisting of one segment. Whether occlusion extended to the vertebral artery (VA) was also analyzed. Recanalization was assessed from controlled angiography images and dichotomized as partial to complete [thrombolysis in myocardial infarction (TIMI) score 2–3] or nil to minimal (TIMI 0–1) (17). Futile recanalization was defined as successful recanalization with no clinical benefit demonstrated as an mRS score 4–6 at 3 months (18).

Collateral vasculature was assessed from baseline angiography images by identifying the presence and number of posterior communicating arteries (PCom) and the presence of at least one superior cerebellar artery (SCA) and posterior cerebral artery (PCA). Symptomatic intracranial hemorrhage (sICH) was defined according to European Cooperative Acute Stroke Study II (ECASS II) criteria (19). Radiological data were analyzed by an experienced neuroradiologist (HS/PV/OS).

### Outcome

The outcome was measured with an mRS obtained at 3 months by a certified neurologist. It was then dichotomized as favorable (mRS 0–3) vs. poor (mRS 4–6). An mRS score of 3 was considered still favorable due to the abysmal natural course of BAO, and this is unlike anterior circulation, where an mRS 0–2 is most often regarded as a favorable outcome. Secondary outcome measures were a good outcome (mRS 0–2) and being bedridden or dead (mRS 5–6) at 3 months. Mortality was also analyzed.

### Statistical Analyses

Dichotomous variables were analyzed with Pearson's chi-square test or Fisher's exact test when appropriate. The distribution of continuous variables was tested for normality and analyses were performed with *t*-test for normally distributed or with Mann-Whitney U-test for non-normally distributed variables. Multivariable analysis of factors associated with coma was made with a stepwise backward logistic regression model, and variables with probability (*p*) value < 0.1 in univariate model were included in order to avoid selection bias and overfitting. For factors associated with favorable outcomes in comatose patients, no multivariable model was possible to construct due to the small number of subjects. Therefore, only the univariate model was built. SPSS 25 (IBM, Armonk, NY, USA) was used in all statistical analyses.

## Results

Among the cohort of 259 BAO patients, 103 (39.8%) presented comatose (GCS ≤8) on admission. 85/103 (82.5%) of comatose patients were intubated. For all of them, the reason for intubation was a poor clinical condition or securing the airway because of impaired consciousness. In total, 35 patients (13.5%) had baseline imaging with MRI, and the remaining majority underwent CT scans. Patient characteristics are shown in Table 1. Comatose patients tended to be older, had more frequent histories of coronary artery disease and myocardial infarctions, and were more likely to present with convulsions. Regarding brain imaging findings of the posterior circulation area, patients with initial coma had more often extensive baseline ischemic lesions as evaluated with pc-ASPECTS < 8 (29.1 vs. 11.6%, *p* < 0.001) and more often radiological signs of bilateral ischemia (35.3 vs. 14.5%, *p* < 0.001).

**Table 1:** Characteristics of comatose vs. non-comatose patients at presentation.

***n* (%)/median (IQR)**	**All (*n* = 259)**	**Non-comatose (*n* = 156, 60.2%)**	**Comatose (*n* = 103, 39.8%)**	***p***
**Demographic and clinical characteristics**				
Male sex	169 (65.3%)	106 (67.9%)	63 (61.2%)	0.262
Age (years)	68 (59–77)	64 (58–75)	73 (63–79)	** <0.001**
Diabetes	42 (16.2%)	24 (15.4%)	18 (17.5%)	0.655
Atrial fibrillation	60 (23.2%)	31 (19.9%)	29 (28.2%)	0.122
Hypertension	149 (57.5%)	85 (54.5%)	64 (62.1%)	0.223
Dyslipidemia	106 (40.9%)	64 (41.0%)	42 (40.8%)	0.968
Chronic heart failure	22 (8.5%)	10 (6.4%)	12 (11.7%)	0.139
Coronary artery disease	46 (17.8%)	21 (13.5%)	25 (24.3%)	**0.026**
History of myocardial infarction	26 (10.0%)	11 (7.1%)	15 (14.6%)	**0.049**
Previous stroke	63 (24.3%)	38 (24.4%)	25 (24.3%)	0.987
Sudden onset phenotype	214 (82.6%)	125 (80.1%)	89 (86.4%)	0.192
Prodromal symptoms	56 (21.6%)	37 (23.7%)	19 (18.4%)	0.313
Convulsions	37 (14.3%)	12 (7.7%)	25 (24.3%)	** <0.001**
**Radiological parameters**				
pc-ASPECTS < 8	48 (18.6%)	18 (11.6%)	30 (29.1%)	** <0.001**
Bilateral ischemia	58 (22.8%)	22 (14.5%)	36 (35.3%)	** <0.001**
Brainstem ischemia	48 (18.9%)	24 (15.8%)	24 (23.5%)	**0.122**
No patent PComs	49 (19.8%)	20 (13.6%)	29 (29.0%)	**0.003**
2 PComs	132 (53.4%)	89 (60.5%)	43 (43.0%)	**0.007**
At least one patent PCA	233 (94.3%)	138 (93.9%)	95 (95.0%)	0.708
At least one patent SCA	172 (69.6%)	114 (77.6%)	58 (58.0%)	**0.001**
Long BAO (≥2 segments)	100 (40.0%)	47 (31.5%)	53 (52.5%)	**0.001**
BAO exceeding to VA	59 (23.7%)	27 (18.1%)	32 (32.0%)	**0.012**
**Treatments**				
IVT	234 (90.3%)	141 (90.4%)	93 (90.3%)	0.980
EVT	75 (29.0%)	45 (28.8%)	30 (29.1%)	0.961
IVT + EVT	53 (20.5%)	32 (20.5%)	21 (20.4%)	0.981
**Time delays (min)**				
OTT	252 (138–720)	238 (125–780)	282 (158–640)	0.334
SDT	155 (78–451)	152 (75–474)	158 (85–371)	0.659
DTN	51 (25–195)	85 (38–180)	65 (30–185)	0.060

*Data available in >95% for all parameters. IQR, interquartile range; pc-ASPECTS, posterior circulation Acute Stroke Prognosis Early CT Score; PCom, posterior communicating artery; PCA, posterior cerebral artery; SCA, superior cerebellar artery; BAO, basilar artery occlusion; VA, vertebral artery; IVT, intravenous thrombolysis; EVT, endovascular treatment; OTT, Onset-to-treatment time, SDT, symptom-to-door time; DTN, Door-to-needle time*.

*Statistically significant p-values (<0.05) are bolded*.

In vascular imaging of the posterior circulation, collateral circulation was less complete in comatose patients as compared to non-comatose patients with absence of patent PComs of the circle of Willis in 29.0 vs. 13.6% (*p* = 0.003) and absence of SCAs in 42.0 vs. 22.4% (*p* = 0.001), respectively. Comatose patients also had multiple segments (≥2) of BA occluded and a higher incidence of occlusions extending to the vertebral artery (VA) (Table 1).

There was no difference in the rate of EVT or IVT between comatose and non-comatose patients, and the vast majority underwent IVT (90.3%). There were only three patients not treated with recanalization therapy. In detail, there was one patient with spontaneous recanalization in DSA and 3-month mRS 3, one patient with EVT attempt but no successful thrombectomy due to difficult stenosis and no access with thrombectomy device (mRS 6), and another with only IVT bolus administered but discontinued infusion due to a history of malignancy (mRS 4). Treatment delays did not differ between comatose and non-comatose BAO patients (Table 1).

In multivariable analysis (Table 2) factors independently associated with acute coma in BAO, patients were of older age, had a history of coronary artery disease, convulsions, presence of extensive baseline ischemia, absence of PComs and SCAs, and long BA occlusion.

**Table 2:** Factors associated with acute coma in BAO patients.

	**OR**	**95% C.I**.	***p***
Age (per year)	1.04	1.01–1.06	0.004
CAD	2.18	1.01–4.74	0.049
Convulsions	4.96	2.12–11.61	<0.001
pc-ASPECTS < 8	3.64	1.74–7.62	0.001
Absence of patent PComs	2.44	1.17–5.09	0.018
At least one patent SCA	0.40	0.21–0.75	0.004
Long BAO (≥2 segments)	2.55	1.39–4.67	0.002

*CAD, coronary artery disease; pc-ASPECTS, posterior circulation Acute Stroke Prognosis Early CT Score; PCom, posterior communicating artery; SCA, superior cerebellar artery; BAO, basilar artery occlusion*.

*Factors with probability value (p) < 0.1 in a univariate model (Table 1) were included in a backward stepwise logistic regression model*.

Initial coma was associated with poor outcome, and 79.6% of comatose patients had an mRS 4–6 at 3 months compared to 38.4% among non-comatose patients (*p* < 0.001). Overall, 59.2% of comatose patients died within 3 months (Table 3), and 72.8% of patients with initial coma were either bedridden or died (mRS 5–6) at 3 months, which compared to the 28.5% of non-comatose patients (*p* < 0.001). Of the comatose patients, 13.6% had an mRS of 5 at 3 months. However, up to 20.4% of comatose patients still had a favorable outcome (mRS 0–3), and 11.7% had a good outcome of mRS 0–2 at 3 months. There was no significant difference in rates of recanalization between comatose and non-comatose patients, but futile recanalization (mRS 4–6) was significantly more common in comatose patients (49.4 vs. 18.5%, *p* < 0.001). There was a trend toward a higher rate of post-treatment sICH in comatose patients (13.9 vs. 7.8%), but this was not statistically significant (*p* = 0.117).

**Table 3:** Outcome measures between comatose vs. non-comatose patients.

**Outcome, *n* (%)**	**All (*n* = 259)**	**Non-comatose (*n* = 156, 60.2%)**	**Comatose (*n* = 103, 39.8%)**	***p***
**90-day mRS**				
mRS 0–2	88 (34.6%)	76 (50.3%)	12 (11.7%)	** <0.001**
mRS 0–3	114 (44.9%)	93 (61.6%)	21 (20.4%)	** <0.001**
mRS 5–6	118 (46.5%)	43 (28.5%)	75 (72.8%)	** <0.001**
Dead at 3 months	98 (38.3%)	37 (24.2%)	61 (59.2%)	** <0.001**
Recanalization (TIMI 2–3)^†^	155 (75.6%)	98 (76.6%)	57 (74.0%)	0.682
Futile recanalization^‡^	61 (30.3%)	23 (18.5%)	38 (49.4%)	** <0.001**
sICH	26 (10.2%)	12 (7.8%)	14 (13.9%)	0.117

† *Data available in 79%*.

‡ *Data available in 78%*.

*mRS, modified Rankin Scale; TIMI, Thrombolysis in Myocardial Infarction; sICH, symptomatic intracerebral hemorrhage*.

*The outcome was assessed at 3 months. Futile recanalization was determined as successful recanalization but ending up with a poor outcome (modified Rankin Scale 4–6). Symptomatic intracranial hemorrhage (sICH) was defined according to European Cooperative Acute Stroke Study II (ECASS II) criteria*.

*Statistically significant p-values (<0.05) are bolded*.

An exact GCS score was available for 83/103 of comatose patients. A total of 19 patients had GCSs scores of three. The GCS was four in 18 patients, five in 15 patients, six in 11 patients, and seven in 14 patients. Only six patients had GCS score of eight. In total, 3/19 (15.8%) of patients with a GCS score of three had an mRS 0–3 at 3 months. The rest of these patients (16/19, 84.2%) had the poorest outcome of an mRS of 5–6, and 13 of them (68.4%) were dead at 3 months. Half of the patients with GCS score of eight had an mRS 0–3 at 3 months, one died, and two had the poorest outcome (mRS 5–6).

Characteristics associated with favorable outcomes in patients with initial coma are shown in Table 4. Patients with favorable 3-month outcome were younger [median age 65 (IQR 57–74) vs. 74 (66–80) years, *p* = 0.010] and displayed less frequent extensive baseline ischemic lesions (pc-ASPECTS < 8 in 9.5 vs. 34.1%, *p* = 0.027) compared with patients with an outcome mRS of 4–6. Other demographic or clinical characteristics did not differ between the outcomes of comatose patients, nor did the radiological surrogates of vascular collateralization. Recanalization was a necessary requirement for a favorable outcome (i.e., mRS 0–3) in all but two patients (in 19/21). None of the patients that achieved a favorable outcome had sICH (Table 4).

**Table 4:** Characteristics associated with favorable outcome in comatose BAO patients.

***n* (%)/median (IQR)**	**All (*n* = 103)**	**mRS 0–3 (*n* = 21, 20.4%)**	**mRS 4–6 (*n* = 82, 79.6%)**	***p***
**Characteristics**				
Male sex	63 (61.2%)	14 (66.7%)	49 (59.8%)	0.562
Age	73 (63–79)	65 (57–74)	74 (66–80)	**0.010**
Diabetes	18 (17.5%)	1 (4.8%)	17 (20.7%)	0.112
Atrial fibrillation	29 (28.2%)	3 (14.3%)	26 (31.7%)	0.113
Hypertension	64 (62.1%)	13 (61.9%)	51 (62.2%)	0.980
Dyslipidemia	42 (40.8%)	6 (28.6%)	36 (43.9%)	0.202
Chronic heart failure	12 (11.7%)	4 (19.0%)	8 (9.8%)	0.259
Coronary artery disease	25 (24.3%)	5 (23.8%)	20 (24.4%)	0.956
Previous myocardial infarction	15 (14.6%)	3 (14.3%)	12 (14.6%)	1.000
Previous stroke	25 (24.3%)	4 (19.0%)	21 (25.6%)	0.531
Sudden onset phenotype	89 (86.4%)	18 (85.7%)	71 (86.6%)	1.000
Prodromes	19 (18.4%)	3 (14.3%)	16 (19.5%)	0.757
Convulsions	25 (24.3%)	5 (23.8%)	20 (24.4%)	0.956
Lowest GCS on admission^†^	5 (4–6)	6 (4–7)	5 (4–6)	0.212
**Radiological parameters**				
pc-ASPECTS < 8	30 (29.1%)	2 (9.5%)	28 (34.1%)	**0.027**
Bilateral ischemia	36 (35.3%)	5 (23.8%)	31 (38.3%)	0.217
Brainstem ischemia	24 (23.5%)	3 (14.3%)	21 (25.9%)	0.388
No patent PComs	29 (29.0%)	6 (30.0%)	23 (28.8%)	0.912
2 PComs	43 (43.0%)	10 (50.0%)	33 (41.3%)	0.480
At least one patent PCA	95 (95.0%)	19 (95.0%)	76 (95.0%)	1.000
At least one patent SCA	58 (58.0%)	11 (55.0%)	47 (58.8%)	0.761
Long BAO (≥2 segments)	53 (52.5%)	11 (55.0%)	42 (51.9%)	0.801
BAO exceeding to VA	32 (32.0%)	4 (20.0%)	28 (35.0%)	0.198
Recanalization (TIMI 2–3)^‡^	57 (74.0%)	19(95.0%)	38 (66.7%)	**0.013**
sICH (ECASS II)	14 (13.9%)	0 (0.0%)	14 (17.5%)	**0.038**
**Treatments**				
IVT	93 (90.3%)	19 (90.5%)	74 (90.2%)	0.974
EVT	30 (29.1%)	5 (23.8%)	25 (30.5%)	0.548
IVT + EVT	21 (20.4%)	3 (14.3%)	18 (22.0%)	0.554
**Time delays (min)**				
OTT	282 (158–640)	247 (165–415)	305 (158–640)	0.595
SDT	158 (85–371)	170 (106–371)	156 (82–371)	0.964
DTN	85 (38–180)	89 (51–165)	85 (38–189)	0.902

† *Data available in 81%*.

‡ *Data available in 75%*.

*mRS, modified Rankin Scale; IQR, interquartile range; GCS, Glasgow Coma Scale; pc-ASPECTS, posterior circulation Acute Stroke Prognosis Early CT Score; PCom, posterior communicating artery; PCA, posterior cerebral artery; SCA, superior cerebellar artery; BAO, basilar artery occlusion; VA, vertebral artery; TIMI, thrombolysis in myocardial infarction; IVT, intravenous thrombolysis; EVT, endovascular treatment; OTT, Onset-to-treatment time; SDT, symptom-to-door time; DTN, Door-to-needle time*.

*Univariate model; a multivariable regression model was not constructed due to the small sample size*.

*Statistically significant p-values (<0.05) are bolded*.

Factors predicting the worst possible outcome, projected being bedridden or dead, in a univariate model are shown in Table 5. Patients with a 3-month mRS 5–6 were older, presented with diabetes and atrial fibrillation more frequently, presented with lower median GCS score [median 4 (IQR 3–6) vs. 6 (5–7), *p* = 0.026], and had more often extensive baseline ischemic changes and bilateral ischemia in baseline imaging. All patients with acute coma and post-treatment sICH had an mRS 5–6 at 3 months (11 dead and 3 with an mRS of 5). The rate of recanalization was lower in patients with the worst outcome (64.0 vs. 88.9%, *p* = 0.006). Extensive baseline ischemic changes were a major factor predicting the poorest outcome (mRS 5–6). In total, 30/103 (29.1%) of the acutely comatose patients had pc-ASPECTS < 8, and only two of them achieved favorable outcomes (mRS 0–3). In terms of patients with acute coma and pc-ASPECTS < 8, 93.3% (28/30) were bedridden or dead (mRS 5–6) after 3 months (Table 5). When patients with pc-APECTS < 8 were excluded from analysis, 26.0% (19/73) of patients with initial coma achieved favorable outcome, and 64.4% (47/73) had an mRS 5–6.

**Table 5:** Factors associated with poorest outcome (mRS 5–6) in comatose BAO patients.

***n* (%)/mean (SD)/median (IQR)**	**All (*n* = 103)**	**mRS 0–4 (*n* = 28, 27.2%)**	**mRS 5–6 (*n* = 75, 72.8%)**	***p***
**Characteristics**				
Male sex	63 (61.2%)	19 (67.9%)	44 (58.7%)	0.395
Age	71 (12)	65 (14)	73 (10)	**0.001**
Diabetes *n*	18 (17.5%)	1 (3.6%)	17 (22.7%)	**0.022**
Atrial fibrillation	29 (28.2%)	3 (10.7%)	26 (34.7%)	**0.016**
Hypertension	64 (62.1%)	18 (64.3%)	46 (61.3%)	0.783
Dyslipidemia	42 (40.8%)	9 (32.1%)	33 (44.0%)	0.276
Chronic heart failure	12 (11.7%)	4 (14.3%)	8 (10.7%)	0.731
Coronary artery disease	25 (24.3%)	7 (25.0%)	18 (24.0%)	0.916
Previous myocardial infarction	15 (14.6%)	3 (10.7%)	12 (16.0%)	0.754
Previous stroke	25 (24.3%)	6 (21.4%)	19 (25.3%)	0.681
Sudden onset phenotype	89 (86.4%)	25 (89.3%)	64 (85.3%)	0.753
Prodromes	19 (18.4%)	4 (14.3%)	15 (20.0%)	0.506
Convulsions	25 (24.3%)	7 (25.0%)	18 (24.0%)	0.916
Lowest GCS on admission^†^	5 (4–6)	6 (5–7)	4 (3–6)	**0.026**
**Radiological parameters**				
pc-ASPECTS < 8	30 (29.1%)	2 (7.1%)	28 (37.3%)	**0.003**
Bilateral ischemia	36 (35.3%)	5 (17.9%)	31 (41.9%)	**0.023**
Brainstem ischemia	24 (23.5%)	3 (10.7%)	21 (28.4%)	0.061
No patent PComs	29 (29.0%)	9 (33.3%)	20 (27.4%)	0.561
2 PComs	43 (43.0%)	13 (48.1%)	30 (41.1%)	0.527
At least one patent PCA	95 (95.0%)	25 (92.6%)	70 (95.9%)	0.610
At least one patent SCA	58 (58.0%)	14 (51.9%)	44 (60.3%)	0.449
Long BAO (≥2 segments)	53 (52.5%)	14 (51.9%)	39 (52.7%)	0.940
BAO exceeding to VA	32 (32.0%)	6 (22.2%)	26 (35.6%)	0.202
Recanalization (TIMI 2–3)^‡^	57 (74.0%)	25 (92.6%)	32 (64.0%)	**0.006**
sICH (ECASS II)	14 (13.9%)	0 (0.0%)	14 (19.2%)	**0.010**
**Treatments**				
IVT	93 (90.3%)	26 (92.9%)	67 (89.3%)	0.724
EVT	30 (29.1%)	8 (28.6%)	22 (29.3%)	0.940
IVT + EVT	21 (20.4%)	6 (21.4%)	15 (20.0%)	0.873
**Time delays (min)**				
OTT	282 (158–640)	235 (162–379)	315 (158–642)	0.237
SDT	158 (85–371)	159 (98–368)	158 (82–451)	0.961
DTN	85 (38–180)	69 (38–127)	89 (38–214)	0.216

† *Data available in 81%*.

‡ *Data available in 75%*.

*mRS, modified Rankin Scale; SD, Standard deviation; IQR, interquartile range; GCS, Glasgow Coma Scale; pc-ASPECTS, posterior circulation Acute Stroke Prognosis Early CT Score; PCom, posterior communicating artery; PCA, posterior cerebral artery; SCA, superior cerebellar artery; BAO, basilar artery occlusion; VA, vertebral artery; TIMI, thrombolysis in myocardial infarction; IVT, intravenous thrombolysis; EVT, endovascular treatment; OTT, Onset-to-treatment time; SDT, symptom-to-door time; DTN, Door-to-needle time*.

*Univariate model; a multivariable regression model was not possible to construct due to the small sample size*.

*Statistically significant p-values (<0.05) are bolded*.

## Discussion

**Figure 2:**
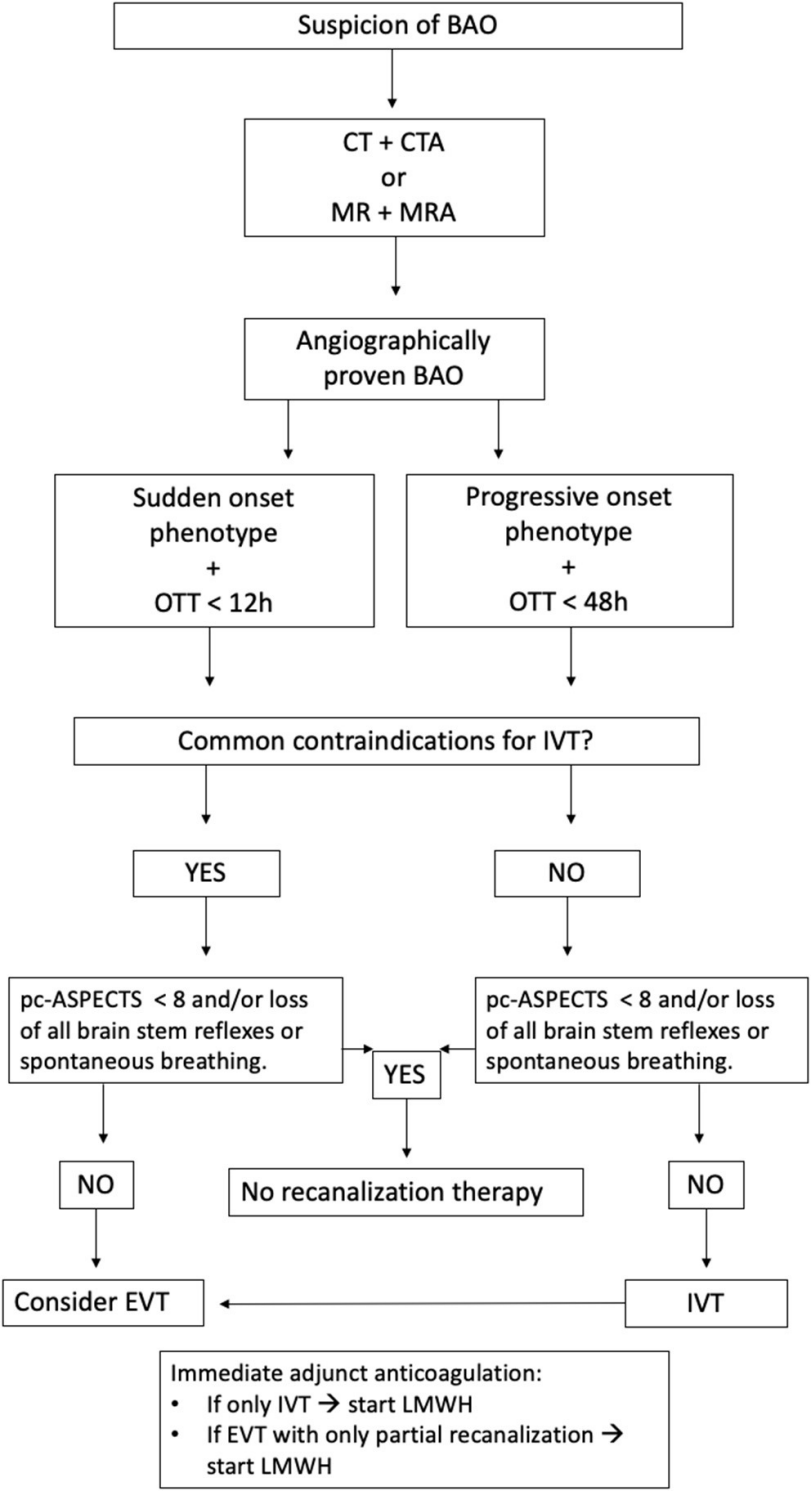
Simplified flow chart on BAO treatment. BAO, Basilar artery occlusion; OTT, Onset-to-teatment time; IVT, Intravenous thrombolysis; pc-ASPECTS, posterior circulation Acute Stroke Prognosis Early CT Score; EVT, Endovascular treatment; LMWH, Low-molecular-weight heparin.

Approximately 40% of BAO patients were initially comatose, which is in line with rates of 30–60% reported in previous studies (3–10). The main observation of this study was that, despite the dismal presentation on admission, still 2/10 comatose BAO patients treated with recanalization therapy reached functionally meaningful survival. Coma vs. no coma on presentation was not associated with differences in receiving either IVT or EVT treatment, nor with the differences in onset to treatment or symptom to door times. Our data consists of a large cohort of BAO patients treated in our institution between 1995 and 2019. In view of data from RCTs published in 2015 (20) demonstrating the efficacy of EVT in large vessel occlusions in anterior circulation, EVT was adopted in more routine use only after this in 2015. As previously reported by us (21), our IVT-treated cohort had comparable outcome rates with many EVT cohorts, and therefore no evidence-based amendment toward EVT-based treatment strategy has been made in our written in-house stroke management guidelines (Figure 2), although the rates of EVT have increased in recent years.

In our present cohort, older age, a history of coronary artery disease as a manifestation of atherosclerosis, convulsions at onset, extensive early ischemia in the vertebrobasilar region, and a lack of patent collateral vasculature of either circle of Willis or cerebellum, besides long occlusions, were independently associated with acute coma in BAO patients. All of these have a logical neuroanatomical and neurophysiological explanation. Anatomically, the area vital to upholding consciousness is the pontine tegmentum, containing the ascending reticular activating system with a complex network of neurons (1, 11, 22, 23). Cerebrovascular atherosclerosis often leads to occlusions of the proximal and middle segments of the BA resulting in pontine ischemia and affecting areas upholding consciousness (1). Convulsive-like movements and extension rigidity arise from pontine pyramidal tracts and are therefore suggestive of brainstem ischemia often involving also areas of reticular activating circuitry (1, 2). Posterior circulation (Pc) ASPECTS below 8 correlates well with the brainstem ischemia since 64.6% of patients with pc-ASPECTS < 8 had brainstem ischemia either alone or combined with other VB-areas.

The observation that multisegmental occlusions of BA associated with initial coma are consistent with the probability of a greater number of perforating pontine arteries lacks patency on presentation. In line with these findings, both Ferbert et al. (3) and Cross et al. (24) reported a clot location most often in either proximal or middle segment of BA in comatose BAO patients. The finding that collateral status associated with coma on presentation is consistent with the concept of reverse “backflow” from the PComs and SCAs supplying circulation in the arterial branches distal to BAO maintaining brainstem vitality for longer periods of time (25). Greater collateral status has been reported to be associated with a better prognosis (26, 27).

The rates of 35 and 45% of good (mRS 0–2) and favorable outcomes (mRS 0–3), respectively, among the whole BAO cohort (Table 3) are in line with or slightly better than in previous cohorts (10, 12, 14, 18, 27–30). Poorer outcomes were significantly more frequent in comatose patients (Table 3). Multiple previous studies report unconsciousness as a predictor of poor outcome (6, 12, 14, 31–33), while others have failed to demonstrate such correlation reaching statistical significance (4, 5, 9, 10, 24). This might be in part explained by the small number of study subjects and varying patient selection. When ischemic stroke patients requiring ventilation support were analyzed, GCS < 10 on admission was an independent predictor of both early and late mortality (34). However, in the present study favorable outcome (3-month mRS 0–3) was achieved in one-fifth of BAO patients with initial coma. Similar results have been previously reported in the BASICS registry with a rate of good outcome of 17% in intraarterial thrombolysis (IAT) and 26% in IVT treated BAO patients with coma, tetraplegia, or locked-in state on presentation (13). In our previous smaller study, we found a favorable outcome (mRS 0–3) in 22.5% of BAO patients needing intubation and mechanical ventilation (14). These rates are slightly higher than those reported by Pallesen et al. (6) with 15.4% of comatose BAO patients achieving a favorable outcome (mRS 0–3). This might be partly explained with a relatively high number of patients with no recanalization therapy in their study since 33% of comatose patients did not receive any recanalization therapy, 38% underwent IAT, 4% were treated with IVT, and 24% were treated with IVT + IAT.

Since reduced consciousness in the case of BAO arises from brainstem ischemia that might be reversed with rapid recanalization therapy, it is conceivable that even a patient with acute coma can be recovered if permanent brainstem tissue damage remains limited. Indeed, Chandra and coworkers (4) reported that low GCS scores did not correlate with poor neurologic outcomes in patients with acute BAO managed with intraarterial therapy (4). Furthermore, Nagel et al. (9) reported that lower GCS was associated with less favorable outcome but when the multivariable model was construed, only pc-ASPECTS remained as an independent predictor of outcome (9). Similarly, 96% of patients with pc-ASPECTS < 8 at baseline had poor outcomes (3-month mRS 3–6) in our previous study (35). In the present study, 93% of patients with acute coma and pc-ASPECTS < 8 ended up dead or bedridden at 3 months. With further simulation of the current cohort, it was observed that, when BAO patients with low pc-ASPECTS were excluded from the analysis, the rate of favorable outcome raised to 26% within comatose patients, and the rate of the most devastating outcome of mRS 5–6 reduced to 64% (vs. 72.8%). This supports our present in-house treatment guideline in which extensive brainstem ischemia is a contraindication for BAO recanalization treatment but not coma as such (Figure 2). Since the cohort includes patients also from a period prior to the European label for IVT use in acute stroke, the experience and adherence to in-house protocol for BAO treatment has improved over the years (21).

Besides the absence of ischemic changes, factors associated with favorable outcomes in comatose BAO patients included younger age, successful recanalization, and avoiding post-treatment sICH, the latter two of which are treatment-related efficacy and safety goals worth further honing. On the other hand, additional factors associated with the poorest outcome (mRS 5–6) were older age, a burden of cardiovascular comorbidities (i.e., diabetes and atrial fibrillation), being deeply comatose, and imaging showing extensive ischemic changes. Although several of these factors have previously been shown to predict outcome after BAO (12, 14, 28–30, 36, 37), the relative predictive value of these factors projecting poor and favorable outcomes of initially comatose patients has not been described in detail due to relatively small study populations and cohort sizes of BAO. We remain with the clinical challenge of weighing each of these in decision making, taking all of them into consideration in each BAO patient.

An acutely comatose BAO patient is one of the most challenging emergencies in neurological ED evaluation. After the correct diagnosis has been reached, physicians need to conduct risk stratification to support decision making about the aggressiveness of the immediate therapy. The prognosis of BAO without recanalization is abysmal (1, 2), but the rate of futile recanalization is also high; roughly 50% of comatose patients in the present study did not avoid institutionalized living conditions or death despite success in recanalization. Mortality among mechanically ventilated stroke patients is known to be high, yet some of these patients do survive, occasionally even with no or only slight disability (15). It is known that physicians tend to be overly pessimistic in predicting survival and quality of life in patients with severe stroke or other critical illness and that many physicians' personal mindsets also affect their treatment decisions (15). This should be acknowledged in order to avoid a self-fulfilling prophecy. On the other hand, novel endovascular recanalization procedures and life-sustaining therapies in intensive care and stroke units are considerably expensive when given futilely to reach excellent vascular patency in patients projected toward a desperate prognosis. Avoiding sheer mortality should not be the steadfast main goal, and the probability of meaningful recovery with an acceptable quality of life should always be kept in mind when making treatment decisions. Therefore, every evidence-based assistance available is invaluable for physicians treating these infrequent BAO patients. This underlines the importance of our present evaluation of factors that might facilitate the challenging risk stratification and decision making in the acute setting.

Limitations of the present study are the relatively restricted number of subjects and the incomplete availability of radiological recanalization data for all patients, especially the ones with early clinical deterioration or death within 24 h. Due to the small number of comatose patients, no multivariable regression models regarding their outcome were possible to construe. However, we report data from a previously well-established single-center cohort of an experienced stroke unit with a long-honed recanalization therapy protocol for BAO (5, 7, 14, 21, 30, 35, 38).

In conclusion, one-fifth of BAO patients presenting with initial coma achieved a favorable outcome within 3 months. These patients were younger and lacking extensive baseline ischemic lesions of the vertebrobasilar region on baseline brain imaging. Therefore, unconsciousness *per se* and seemingly bleak clinical conditions upon presentation should not as such exclude BAO patients from receiving aggressive recanalization therapy. Rather, initial and post-treatment decisions during subsequent days should be made individually, considering prognostic key baseline characteristics, where the volume and location of infarction should be dominant in assisting maintenance or de-escalation of intensive care (9, 39). These characteristics can be brought up in discussions with the closest ones and when making decisions regarding the prolongation of intensive care unit treatment.

## Data Availability Statement

The original contributions presented in the study are included in the article/supplementary material, further inquiries can be directed to the corresponding author/s.

## Author Contributions

JR, TS, PL, and DS contributed to the design of the study and the writing process of the manuscript. HS, OS, and PV collected the radiological data. JR performed statistical analyses. All authors contributed to manuscript revision, read, and approved the submitted version.

## Conflict of Interest

The authors declare that the research was conducted in the absence of any commercial or financial relationships that could be construed as a potential conflict of interest.
